# Long pentraxin 3 and vitamin D receptor mRNA expression pattern of cumulus granulosa cells isolated from PCOS oocytes at different stages of nuclear maturation

**DOI:** 10.1186/s12958-023-01176-5

**Published:** 2024-01-02

**Authors:** Aynur Ersahin, Onder Celik, Nur D. Gungor, Nilufer Celik, Sureyya Melil, Meltem Yardim, Semih Dalkilic, Cenk Ersahin, Ece Dogukargin, Sudenaz Celik, Ramazan F. Akkoc

**Affiliations:** 1 Department of Obstetrics and Gynecology, Bahcesehir University Goztepe Medicalpark Hospital, Istanbul, 34732 Turkey; 2 Department of Obstetrics and Gynecology, Private Clinic, Usak, 64000 Turkey; 3 Department of Medical Biochemistry, Behcet Uz Children’s Hospital, Izmir, 35210 Turkey; 4 Göztepe Medicalpark Hospital IVF-Unit, Istanbul, 34732 Turkey; 5 Department of Medical Biochemistry, Yerkoy State Hospital, Yozgat, 66900 Turkey; 6 Faculty of Science, Department of Biology, Molecular Biology and Genetics Program, Firat University, Elazig, Turkey; 7 Bahcesehir University School of Medicine, Istanbul, 34732 Turkey; 8 Medical Faculty, Sofia University “St. Kliment Ohridski”, Sofia, 1407 Bulgaria; 9 Department of Anatomy, School of Medicine, Firat University, Elazig, 34060 Turkey

**Keywords:** Polycystic ovary syndrome, Cumulus expansion, VDR, PTX3, fertilization

## Abstract

**Background:**

A fine-tuned pro-inflammatory and anti-inflammatory balance in the follicular unit is essential for cumulus expansion and successful ovulation. While the long pentraxin 3 (PTX3) gene is required for the expansion of cumulus cells (CCs), ovulation, resumption of meiosis and fertilization, the vitamin D receptor gene (VDR-X2) is required for intra-follicle redox balance. This study was planned to determine the expression pattern of VDR-X2 and PTX3 mRNA in CCs isolated from germinal vesicle (GV), metaphase I (MI), and metaphase II (MII) oocytes of PCOS patients with ovulatory dysfunction.

**Methods:**

The relative expression of CC-PTX3 and CC-VDR-X2 mRNA were evaluated using qRT-PCR in a total of 79 CC samples collected from individual cumulus-oocyte complex of 40 infertile patients (20 PCOS and 20 non-PCOS normal responders) who underwent ovarian stimulation with the GnRH antagonist protocol.

**Results:**

Relative PTX3 mRNA expressions of CCMI-control and CCMII-control showed 3- and 9-fold significant upregulation compared to CCGV-control, respectively. The relative PTX3 mRNA expression of CCMII-control increased approximately three fold compared to CCMI-control. Compared to CCGV-pcos, a 3-fold increase was noted in the relative PTX3 mRNA expression of CCMI-pcos and an approximately 4-fold increase in the PTX3 mRNA expression of CCMII-pcos. Relative PTX3 mRNA expression values of CCMII-pcos and CCMI-pcos were similar. A 6-fold upregulation of relative PTX3 mRNA and a 4-fold upregulation of VDR-X2 mRNA were detected in CCMII-control compared to CCMII-pcos. CC-VDR-X2 expression patterns of the PCOS and control groups overlapped with the CC-PTX3 pattern. Fertilization rates of the PCOS group exhibiting failed transcript expression were similar to normal responders.

**Conclusion:**

The fact that relative CC-PTX3 and CC-VDR mRNA expression does not increase during the transition from MI to MII stage in PCOS as in normal responders suggests that PTX3 and VDR expression may be defective in cumulus cells of PCOS patients with ovulatory dysfunction.

## Background

The ovarian follicular unit is a structure formed by the oocyte and granulosa cells and is responsible for the gradual acquisition of oocyte developmental competence both during folliculogenesis and after ovulation [1–3]. The somatic cells surrounding the oocyte are called cumulus cells (CCs) and those surrounding the antrum are called mural granulosa cells (GCs). The differentiation of both cell types mainly occur under the influence of different growth factors originating from the oocyte, as well as gonadotrophins and IGF-I [2]. The heterologous gap junctions between the CCs and the oocyte allow bidirectional communication to complete the nuclear and cytoplasmic maturation of the oocyte [4–6]. Growth differentiation factor-9 (GDF-9) and bone morphogenetic protein 15 (BMP-15) secreted by the oocyte reach the cumulus cells and regulate their functions [7–9]. CCs are embedded in an extracellular matrix (ECM) rich in hyaluronic acid [7, 10]. The structural content of the cumulus ECM and the expansion of CCs are critical for successful ovulation, resumption of meiosis, tubal transport of cumulus–oocyte complexes (COCs) and fertilization [10]. Different endocrine, metabolic and paracrine inputs enable CCs expansion by remodeling the cumulus cell ECM [9]. It is imperative to maintain hemostasis between pro- and anti-inflammatory cytokines for the cumulus expansion to occur by preventing uncontrolled proinflammatory conditions. One of the anti-inflammatory genes responsible for cumulus expansion is long pentraxin 3 (PTX3) [11, 12]. PTX3 is a potential oocyte quality biomarker expressed in human cumulus cells and associated with cumulus cell expansion [11–14]. It is the target gene of oocyte-derived GDF-9 and is critical for oocyte developmental competence [13]. Defective expression of PTX3 causes failure of both cumulus expansion and in vivo fertilization [13, 14].

Polycystic ovary syndrome (PCOS) is a metabolic disease with low grade inflammation characterized by a defect in conversion of small antral follicles to mature follicles [15]. Ovulatory dysfunction (OD) is considered the most common cause of infertility and is thought to be a cumulus expansion defect in its etiology [7, 16]. Low-grade chronic inflammation in PCOS can cause both follicular maturation defect and anovulation by altering the inflammatory balance required for cumulus expansion and ovulation [17]. Consistent with this, it has been shown that the expression of genes and matrix proteins responsible for cumulus expansion in the granulosa cells of PCOS patients are defective [7]. It has also been reported that women with PCOS have decreased PTX3 expression in blood and CCs [18–20]. The demonstration of a long non-coding RNA expression defect in microarray analysis of CCs from PCOS patients is further evidence supporting the role of the CC expansion defect in PCOS-induced ovulatory dysfunction [21].

Although PTX3 is critical in maintaining the intrafollicular pro-anti-inflammatory cytokine balance, many different genes such as prostaglandin-endoperoxide synthase-2 (COX2), gremlin 1, urokinase-type plasminogen activator and hyaluronic acid synthase 2 may play a role in maintaining this balance [8, 9, 22, 23]. One of the most important candidates in the ever-growing CC expansion gene list is the vitamin D receptor (VDR) gene. In addition to hyperandrogenemia and high central fat deposition, low serum vitamin D (VD) levels may also play a role in the etiology of low-grade chronic inflammation in PCOS [16, 24]. The fact that serum VD levels are reported to be low in most studies in PCOS suggests that this secosteroid hormone and its receptor may play a role in ovulatory dysfunction and cumulus expansion defect [25, 26]. Indeed, VDR is widely expressed not only in tissues involved in calcium metabolism but also in CCs [27]. Binding of VDR in CCs with active VD contributes to the flawless functioning of folliculogenesis by stimulating the production of 3β-hydroxysteroid dehydrogenase (3β-HSD), progesterone, estradiol, estrone, and insulin-like growth factor binding protein-1 [28]. Due to all these effects, VD may contribute to the inflammatory balance required for cumulus enlargement by exhibiting anti-inflammatory and immunomodulatory effects through CC-VDR [29]. Despite isolated studies investigating CC-PTX3 expression in PCOS [14, 18], there is no study investigating the CC-VDR mRNA expression pattern in PCOS patients undergoing controlled ovarian stimulation (COS). This study was, therefore, planned to determine the expression pattern of VDR and PTX3 mRNA in CCs isolated from germinal vesicle (GV), metaphase I (MI), and metaphase II (MII) oocytes of PCOS patients with ovulatory dysfunction.

## Methods

### Study design

Following the approval of the ethics committee (Ethical approval number: 2023/06–07), forty infertile patients whose informed consent was obtained were included in the study. Cumulus cells (CCs) were collected from PCOS (n = 20) and non-PCOS normal responders (n = 20) who underwent ICSI following controlled ovarian stimulation with the GnRH antagonist protocol with GnRH agonist trigger. Women who met at least two of the criteria for hyperandrogenemia, ovulatory dysfunction and polycystic ovarian morphology determined by the European Society for Human Reproduction and Embryology/American Society for Reproductive Medicine (ESHRE/ASRM) were diagnosed with PCOS [30]. While patients in the PCOS group were selected from phenotypes A, B and D with ovulatory dysfunction component, phenotype C without ovulatory dysfunction was excluded from the study. Participants in the control group were selected from patients with male factor infertility who did not have clinical and laboratory findings of PCOS and responded normally to COS. The mean age, BMI, and menstrual history of the participants in both groups were recorded. Endometrial thicknesses were recorded on the day of egg retrieval of both groups. Each partner was subjected to semen analysis according to the World Health Organization 2010 normative reference values.

The common inclusion criteria for participants in both the PCOS and non-PCOS groups included women undergoing antagonist protocol with agonist trigger, aged: 20–35 years, BMI: 18.5–29.9 kg/m2, and embryos: total freezing. Those who used hCG for the ovulation trigger were not included in the PCOS or control group. Exclusion criteria were as follows: type 2 diabetes, thyroid pathology, hyperprolactinemia, congenital adrenal hyperplasia, Cushing’s syndrome, use of insulin sensitizers, lipid-lowering or other hormonal drugs in the past three months. Those undergoing uni- or bilateral ovarian drilling, mecahnical endometrial injury, ovarian cystectomy or salpingectomy were also excluded. Baseline hormone parameters were measured in the 2–5 days of progesterone withdrawal bleeding in the PCOS participants and in the spontaneous menstrual bleeding in normal responders. Serum 25-hydroxyvitamin D, luteinizing hormone (LH), follicle stimulating hormone (FSH), estradiol, insulin, and total testosterone levels were analysed with chemiluminescence immunoassay method in Cobas e602 analyzer (Roche Diagnostics GmbH, Mannheim, Germany) in blood samples collected after 8–10 h of over night fasting. Homeostasis model assessment of insulin resistance (HOMA)-IR was calculated with the following formula: insulin (µU/mL) x glucose (mg/dL)/405 [31].

Both groups underwent controlled ovarian stimulation with flexible GnRH antagonist protocol [32]. Recombinant FSH treatment (Gonal-f, Merck-Serono, Italy) was started on the third day of the spontaneous cycle in the control group and on the third day of spontaneous or progesterone-induced withdrawal bleeding in the PCOS group. The starting dose of gonadotropin was determined by considering the patient’s age, BMI, number of antral follicles and the doses used in previous attempts. When the dominant follicle reached ≥ 14 mm, the pituitary was suppressed with 0.25 mg/day cetrotide (Merck Serono, Switzerland). Ovulation was triggered with 0.2 mg triptorelin acetate when two or more follicles were at least 18 mm in diameter. Ovulation triggering with hCG was not preferred because it could lead to an increase in the expression of cumulus expansion genes [33]. Oocytes were collected 36 h after the ovulation trigger. COCs collected under ultrasound guidance were subjected to cumulus cell processing. Following ICSI, all eligible embryos were frozen. Fertilization rates of each group (2 PN zygotes) were recorded.

### Cumulus cell isolation

CCs were separated from COCs by enzymatic and mechanical methods as previously described [34]. After the aspirated follicle fluids were poured into 90 mm diameter petri dishes, cumulus oocyte complexes (COC) were collected under the microscope and were subjected to the COC classification: COC grade 1 indicates mature oocyte with first polar body (MII stage), COC grade 2 indicates MI stage (absence of GV and first polar body) oocyte, and COC grade 3 indicates GV oocyte. We evaluated cumulus cell morphology during COC grading before denudation in both groups. In the morphological evaluation, the brightness of the cytoplasm, the number of cumulus cell layers, mosaic fragmentation and partial or complete loss of cumulus cells were taken into account. Thus, we had the opportunity to retrospectively compare the changes in CC morphology of groups with different PTX3 or VDR expression. Since oocyte pick-up was first done from follicles of 17–18 mm and above, which were thought to be mature, these COCs were placed in petri dishes labeled grade (1). COCs aspirated from codominant follicles (≥ 15 mm) were placed in petri dishes labeled grade (2). COCs recovered from follicles smaller than 15 mm were placed in plates labeled grade (3). Then, COCs in the petri dishes lifted at a 45-degree angle were evaluated morphologically. The angulation of the petri dish allowed flushing medium to accumulate at the bottom of the petri dish and COCs to adhere to the medium-free area. If COCs adhered to the petri dish and expanded, showed radial protrusion, or polar bodies were visible, they were considered grade 1. Subsequently, if the denudated COCs were compatible with our prediction, they were left in the petri dish labeled grade 1. COCs that did not spread on the petri dish, were dark colored, had a small diameter, or did not have a polar body were considered grade 2 or 3, depending on the diameter of the aspirated follicle. COCs whose post-denudation nuclear development stage did not match grade 1, 2 or 3 were not included in the study. Since it is not possible to see polar body or radial protrusion in every COC subjected to COC grading, an attempt was made to estimate the COC grade according to the experience of our embryologist and the diameter of the aspirated follicle. All COCs were cultured for 2 h prior to denudation (Global total medium, Ref: H5GT-030). At the end of the period, each COC was first exposed to enzymatic denudation using 10 µl of Hyase 10x (Vitrolife, Ref: 10,176), and then mechanically using pipettes with a diameter of 290 µ and 145 µ [35]. The CCs, which were removed from the oocyte by denudation, were transferred to Eppendorf tubes containing RNA-later (ABT, Ref: A100912-L12) and stored at -20 °C until total RNA extraction.

CCs isolated from COCs classified as grade 1 (MII), grade 2 (MI) and grade 3 (GV) were placed in separate vials. CC samples in the PCOS group were labeled as CCGV-pcos, CCMI-pcos, and CCMII-pcos, while vials of non-PCOS patients were labeled CCGV-con, CCMI-con, and CCMII-con (Table 1). The fold changes of VDR and PTX3 mRNA obtained from CCs at different maturation stages were compared both within and between groups. The pooling strategy of biological samples to be subjected to RNA extraction and amplification is not only cost effective, but also increases the sensitivity and specificity for the transcript to be measured [36]. For this reason, we pooled CCs collected from oocytes at the same nuclear development stage in both the PCOS and control groups by placing them in the same vials. A total of 79 pools (42 CC pools from the PCOS group and 37 CC pools from normal responders) were formed, each from oocytes at different nuclear maturation stages. Since sufficient numbers of MII oocytes were obtained from all 20 patients in the PCOS group, a total of 20 pools were formed by placing at least four MII derived CC samples in each pool. Since no MI oocytes could be obtained in 10 of the 20 patients in the PCOS group, at least two or more MI derived CC samples were placed in each pool, forming a total of 10 pools. Since GV oocytes were not detected in eight of 20 PCOS patients, a total of 12 pools, each containing two or more CCs, were formed. Since a sufficient number of MII oocytes were obtained from all 20 patients in the normal responder group, at least four CC samples were placed in each pool, forming a total of 20 pools. Since no MI oocytes could be obtained in 12 of 20 control patients, two or more MI derived CC samples were placed in each pool, forming a total of 8 pools. Since GV oocytes were not detected in 11 of 20 normal responders, a total of 9 pools were formed, each containing two or more CC samples (Table 1).

**Table 1:** CC samples used for RT-qPCR analysis

*Participants*	*COS protocol*	*Oocyte maturation stage*	*Number of pools containing CC*
*PCOS (n = 20)*	GnRH antagonist with agonist trigger	CCGV*-pcos*	12 pools of ≥ 2 samples
		CCMI*-pcos*	10 pools of ≥ 2 samples
		CCMII*-pcos*	20 pools of ≥ 4 samples
*Total number of CC pools*			42
*Non-PCOS (n = 20)*	GnRH antagonist with agonist trigger	CCGV*-con*	9 pools of ≥ 2 samples
		CCMI*-con*	8 pools of ≥ 2 samples
		CCMII*-con*	20 pools of ≥ 4 samples
*Total number of CC pools*			37

### Quantitative reverse transcriptase–polymerase chain reaction

The mRNA expression of the long PTX3 and VDR-X2 mRNA were analyzed using qRT-PCR. Glyceraldehyde-3-Phosphate Dehydrogenase (GAPDH) was preferred as a reference gene when examining mRNA expression. Because human VDR has multiple transcript variants, the receptor structure and cellular response to VD may differ [37]. mRNA variants are created by changing the structure of pre-mRNAs by alternative splicing [38]. We analyzed the VDR-X2 variant. Sequences of all primers designed to be used as forward and reverse primers for qRT-PCR were; PTX3: Forward 5’-TGGACAACGAAATAG ACATGG-3’, Reverse 5’-CTCTCATCTGCGAGTTCTCC-3’, VDR-X2: Forward 5’-ACATTGCTTTGCTTGCCTCC-3’, Reverse 5’-ACGTTCCGGTCAAAGTCTCC-3’, GAPDH: Forward 5’-GAAGATGGTGATGGGATTTC-3’, Reverse 5’-GAAGGTGAAGGTCGGAGTC-3’.

Total RNA isolation from cumulus cells was performed with PureLink Total RNA Mini Kit (Invitrogen) according to manufactures instructions. Qubit Fluorometer (Thermo Fisher, USA) was used to measure RNA concentration. 500 ng RNA was reverse transcribed using the High Capacity cDNA RT Kit (Applied Biosystems, Foster City, CA). Real-time PCR was performed using a StepOne Plus Real Time (Applied Biosystem). Blirt Amplyfyme SYBR Green Master Mix (Cat No: AM-02-200, Gdańsk – Poland) was used in the real-time PCR section. Each real-time PCR sample consisted of 1 µl cDNA, 10 µl 2x AMPLIFYME SG Mix, 0,6 µl of the forward primer, 0,6 µl of the reverse primer, 0,4 µl 50x ROX solution and filled up to 20 µl final volume with PCR grade water. The Real-time PCR program consisted of two steps: 3 min at 95 °C and 40 times for 5 s at 95 °C and then for 30 s at 60 °C. The samples were heated slowly from 72 to 95 °C at the end of the PCR in order to break the DNA and take the melting curve. Housekeeping gene reached the threshold at 24-28th thermal cycles. While CC-PTX3 mRNA of normal responders reached the threshold in the 32-34th thermal cycle, the threshold value for VDR mRNA was in the 34-36th thermal cycle. In the PCOS group, the number of thermal cycles required for both transcripts was 34–38. Since the threshold value to which our device assigned a value, albeit low, was the 40th cycle, values above this limit were not taken into account. The assigned values did not reach the upper limit in any of the samples of normal responders. In the PCOS group, the number of samples approaching the upper limit was approximately 10%, and all the remaining samples reached the threshold in the 37-38th thermal cycles.

### Statistical analysis

IBM SPSS Statistics Version 22.0 for Windows (IBM Corp., Armonk, NY, USA) was used for data analysis. Variables with normal distribution were analyzed by independent sample t-test, and non-normal ones were analyzed with Mann Whitney U test. Continuous variables were given as mean ± standard deviation or median (1st quartile-3rd quartile). Two-tailed *p* values of *p* < 0.05 were considered significant. Expression levels of target genes normalized to GV stage oocytes of patients in the control group were calculated by the ΔΔCt method. Relative gene expression (fold change) was calculated according to the 2^−ΔΔCt^ equation. Whether there was a statistical difference in the expression of PTX-3 and VDR mRNA expression was analyzed by performing two-way ANOVA followed by Bonferroni’s multiple comparisons test with the GraphPad Prism 8.0 (GraphPad Software Inc., San Diego, CA, USA) program.

## Results

The mean age, infertility duration and endometrial thickness measured on the day of egg collection were similar in both groups (Table 2). Serum VD levels of the PCOS group was significantly lower than the control group. Serum LH, total testosterone and HOMA-IR of the PCOS group were significantly higher than the control group. Serum FSH and estradiol values of both groups were similar. The total dose of rFSH consumed in the PCOS group was significantly lower than the control. Serum estardiol values measured on the hCG day and the total number of oocytes collected were significantly higher in the PCOS group compared to the control group.

**Table 2:** Demographic and laboratory findings of PCOS and non-PCOS normal responders

*Variables*	*PCOS (n = 20)*	*Normal responders(n = 20)*	*p-values*
Age (years)	26.95 ± 4.28	27.44 ± 3.51	0.430
BMI (kg/m^2^)	25.12 ± 4.06	22.66 ± 4.69	< 0.001
Infertility duration (yrs)	3 (2–3)	3 (2–3)	0.104
Vitamin D (ng/mL)	12.8 (12–16)	16.1 (15 − 21)	< 0.001
Testosterone (ng/dL)	36.4 (31–38)	17 (16–19.4)	< 0.001
Estradiol (pg/mL)	40.4 ± 3.22	43.6 ± 4.22	0.131
LH (mIU/mL)	8.3 (7–9.5)	5.2 (5–6.3)	< 0.001
FSH (mIU/mL)	5.1 (4–5)	5.2 (4–5)	0.32
HOMA-IR	2.44 ± 0.32	1.39 ± 0.11	< 0.001
Endometrial thickness (mm)	9.5 (9–10)	9.2 (8.7–10)	0.132
Total gonadotropin dose (IU/L)	1623.41 ± 754.3	2649.51 ± 494.6	< 0.001
E2 on the day of hCG (pg/mL)	2870.44 ± 833.7	1965.71 ± 544.8	< 0.001
No. of oocytes retrieved	18.42 ± 5.33	9.51 ± 3.87	< 0.001
MII oocytes	13.25 ± 4,40	5.12 ± 2.08	< 0.001
2 PN zygotes	13/10 (76.9%)	5/4 (80.0%)	0.554

Data are given as mean ± standard deviation or median (1st quartile − 3rd quartile) for continuous variables

**Fig. 1:**
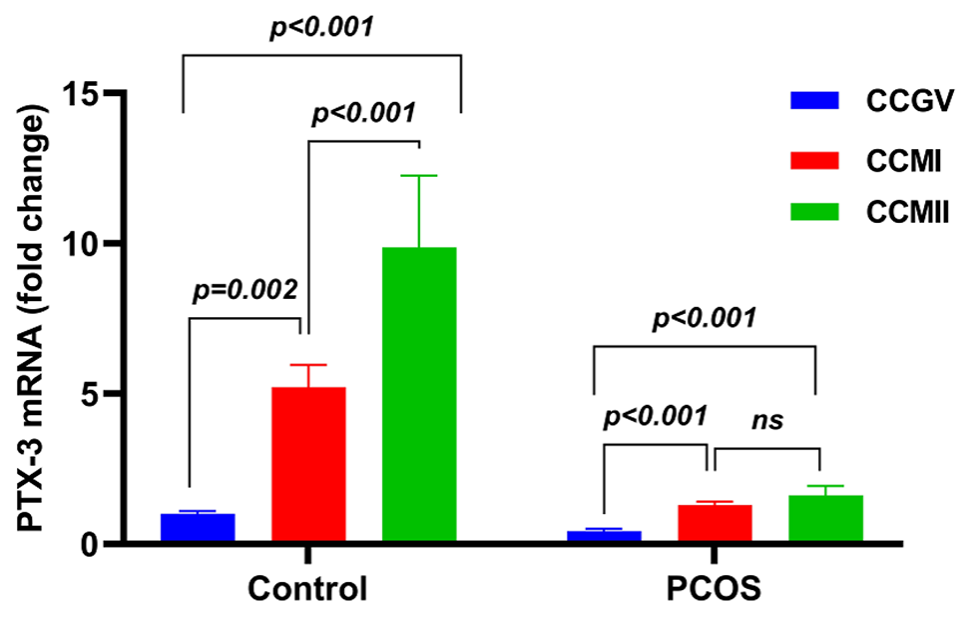
Graphical representation of CC-PTX3 mRNA expression of PCOS and control groups. CC-PTX3 mRNA increases in different nuclear maturation stages of the control group were more pronounced than those in the PCOS group. Note that, unlike the control group, there was no increase in CC-PTX-3 mRNA expression in the PCOS group during the transition between MI and MII. ns: not significant

As shown in Fig. 1; Table 3, the PTX3 mRNA expression of CCMI-control group was 3 times higher than CCGV-control group (*p* < 0.002). PTX3 mRNA expression of the CCMII-control group was 9 times higher than the CCGV-control group (*p* < 0.001). PTX3 expression of the CCMII-con group increased approximately threefold compared to CCMI-con (*p* < 0.001). A 3-fold increase in PTX3 mRNA expression was detected in CCMI-pcos compared to CCGV-pcos (*p* < 0.001). An approximately 4-fold increase in PTX3 expression was noted in CCMII-pcos compared to CCGV-pcos (*p* < 0.001). PTX3 expression change between CCMII-pcos and CCMI-pcos was not significant (*p* < 0.075). The relative PTX3 mRNA value of CCMII-con (9.87 ± 2.38) showed a significant 6.13-fold upregulation compared to CCMII-pcos (1.61 ± 0.31) (*p* < 0.001). Similarly, the relative VDR mRNA value of CCMII-con (11.51 ± 3.22) showed a 4-fold significant upregulation compared to CCMII-pcos (2.87 ± 0.23) (*p* < 0.001).

**Table 3:** Comparison of long PTX3 and VDR mRNA fold changes according to nuclear maturation stage in PCOS and normal responders

*Groups*	*Oocyte nuclear maturation stage*	*PTX3 mRNA* *Fold Change*			*VDR-X2 mRNA* *Fold Change*	
PCOS (n = 20)	1- CCGV-pcos	0.43 ± 0.07			0.68 ± 0.10	
	2- CCMI-pcos	1.29 ± 0.12			2.45 ± 0.32	
	3- CCMII-pcos	1.61 ± 0.31			2.87 ± 0.23	
Normal responders (n = 20)	4- CCGV-con	1.00 ± 0.08			1.03 ± 0.29	
	5- CCMI-con	3.21 ± 0.32			5.63 ± 0.41	
	6- CCMII-con	9.87 ± 2.38			11.51 ± 3.22	
			*p*-values			*p*-values
1 vs. 2	0.43 ± 0.07	1.29 ± 0.12	< 0.001	0.68 ± 0.10	2.45 ± 0.32	< 0.001
1 vs. 3	0.43 ± 0.07	1.61 ± 0.31	< 0.001	0.68 ± 0.10	2.87 ± 0.23	< 0.001
2 vs. 3	1.29 ± 0.12	1.61 ± 0.31	0,075	2.45 ± 0.32	2.87 ± 0.23	0,051
4 vs. 5	1.00 ± 0.08	3.21 ± 0.32	< 0.002	1.03 ± 0.29	5.63 ± 0.41	< 0.007
4 vs. 6	1.00 ± 0.08	9.87 ± 2.38	< 0.001	1.03 ± 0.29	11.51 ± 3.22	< 0.001
5 vs. 6	3.21 ± 0.32	9.87 ± 2.38	< 0.001	5.63 ± 0.41	11.51 ± 3.22	< 0.001
3 vs. 6	1.61 ± 0.31	9.87 ± 2.38	< 0.001	2.87 ± 0.23	11.51 ± 3.22	< 0.001

**Fig. 2:**
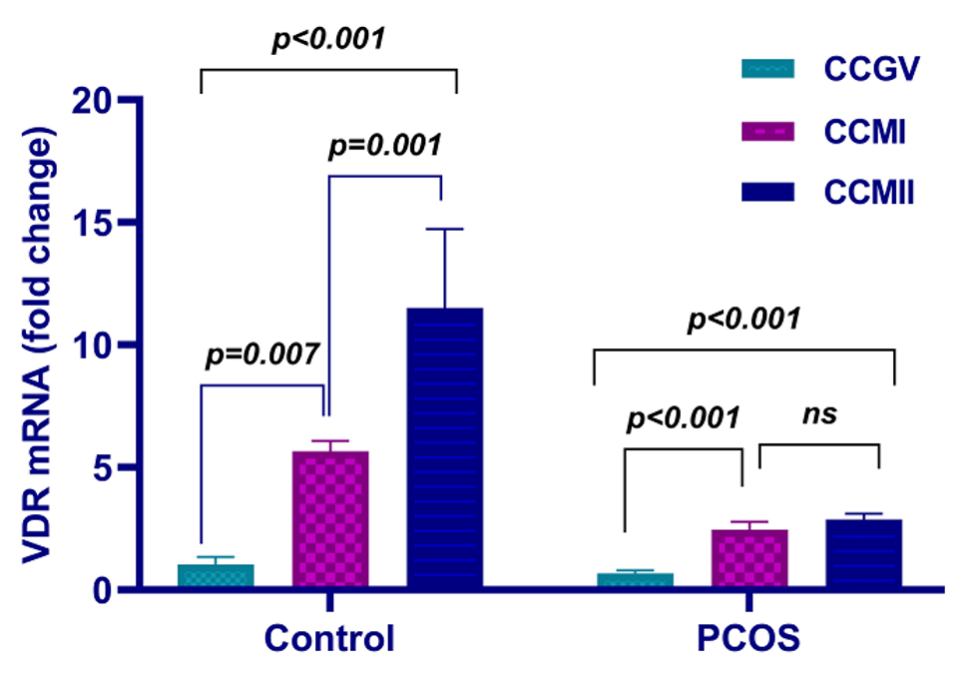
Graphical representation of CC-VDR mRNA expression of control and PCOS groups. CC-VDR mRNA increases in different nuclear maturation stages of the control group were more pronounced than those in the PCOS group. Note that, unlike the control group, there was no increase in CC-VDR mRNA expression in the PCOS group during the transition between MI and MII. ns: not significant

Similar to the PTX3 expression pattern, the increase in VDR-X2 expression between nuclear maturation stages in the PCOS group was less pronounced than in the control group. In contrast to the increase in relative VDR-X2 mRNA expression between MI and MII oocyte transition in the normal responders, the increase in VDR-X2 mRNA expression in MII oocytes in the PCOS group was not different from MI (Fig. 2; Table 3).

**Fig. 3:**
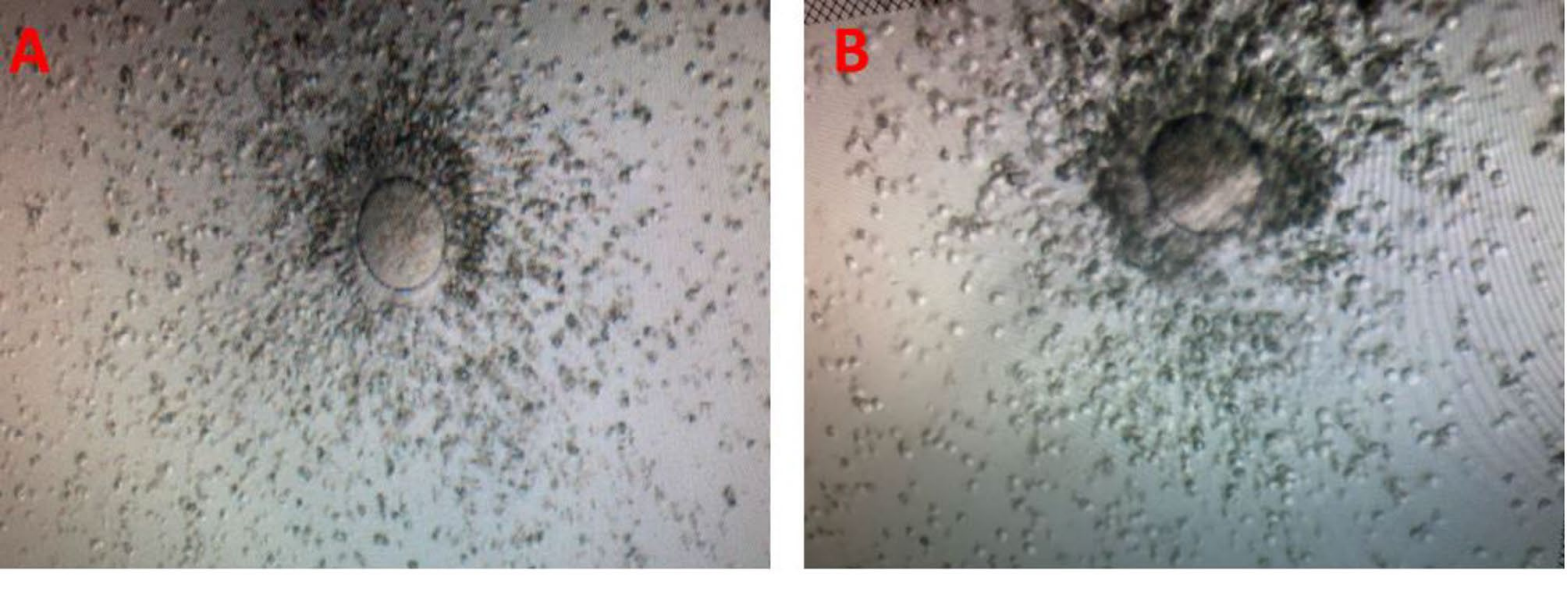
Microscopic view of COC, which was considered grade 1 before denudation and MII oocyte was obtained following denudation (**A**). (**B**) was considered grade 2 before denudation and MI oocyte was obtained after denudation. Morphologically, there was no difference between the PCOS group (**B**), which had low PTX3 and VDR mRNA expression, and the control group (**A**), in which both transcripts were normal, in terms of transparency, the number of cumulus cell layers, mosaic fragmentation and partial or complete loss of cumulus cells. The reason why the COC in B (grade 2) is surrounded by darker and compact cumulus cells than in A (grade 1) is due to the difference in COC grades, not the expression pattern of the cumulus genes. Scale Bars: 100 μm

The 2 PN zygote rate of MII oocytes that underwent ICSI was 76.9% in the PCOS group and 80.0% in the control group. Fertilization rates of the two groups were recorded as similar. Post-ICSI 2PN zygote rates of MII oocytes of PCOS patients expressing low PTX3 and/or VDR were found to be similar to MII oocytes expressing high PTX3 and/or VDR of the control group (Table 2). No significant difference was detected between the COC morphologies of the PCOS group expressing low CC-PTX3 and CC-VDR mRNA and the control group in which both transcripts were expressed normally (Fig. 3).

## Discussion

Ovulatory dysfunction is the most important PCOS component [16] that negatively affects fertility outcome. Since cumulus expansion is critical in ensuring the developmental comeptenence of the oocyte, a defect in the expression of the cumulus expansion genes may cause abnormal development of the oocyte, resulting in adverse fertility outcomes [17]. Hence, expression studies of genes related to cumulus expansion will allow us to advance our ideas and practices regarding oocyte maturation and ovulatory dysfunction in PCOS. Any failed gene expression in cumulus cells may be the cause or result of ovulatory dysfunction in PCOS. It is known that some cumulus expansion genes in PCOS show defective expression [7, 19, 21, 39]. In the current study, we analyzed the expression profiles of VDR and PTX3 mRNA in CCs isolated from GV, MI and MII stage COCs of PCOS and non-PCOS control patients under the GnRH antagonist protocol. Although cumulus cell PTX3 expression has been previously analyzed in PCOS and normal responders [14, 18, 39], CC-VDR mRNA expression of PCOS patients was first analyzed in this study.

We observed a gradual increase in CC-PTX3 mRNA expression from the GV stage oocyte to the MII stage oocyte in non-PCOS normal responders. CC-PTX3 mRNA expression of both MI and MII oocytes of normal responders was significantly higher than GV oocytes. In addition, CC-PTX3 mRNA expression of MII oocytes of the control group was significantly higher than that of MI oocytes. In PCOS group, while CC-PTX3 mRNA expression of MI and MII stage oocytes was significantly higher than GV oocytes, there was no significant difference between MI and MII in terms of PTX3 mRNA expression. The increase in CC-PTX3 mRNA expression between nuclear stages in the PCOS group was less pronounced than in the control group. The lack of change in CC-PTX3 expression in oocytes transitioning from MI stage oocyte to MII suggests that PTX3 expression is defective in PCOS patients. For the oocyte to achieve developmental competence, the CC gene profiles between the stages of nuclear maturation must be different [39]. The proportion of genes differentially expressed in CCs of a MII stage oocyte must be different from both GV and MI stage oocytes [8, 18, 39]. The fact that CC-PTX3 expression was similar in MI and MII stage oocytes in the PCOS group and that the expression intensity of the related gene was lower than in normal responders supports the role of defective CC-PTX3 expression in ovulatory dysfunction. Since intrafollicular pro-anti-inflammatory cytokine balance cannot be achieved in low CC-PTX3 expression, cumulus expansion will be defective in PCOS [8, 9, 22]. Low-grade chronic inflammation in PCOS may trigger cytokine balance towards inflammation, leading to deterioration of follicular dynamics and defects in PTX3 expression and cumulus expansion [40]. In line with this, failed CC-PTX3 expression may lead to inadequate cumulus expansion and oligo-anovulatory cycles, preventing the oocyte from reaching developmental competence. However, the similarity of ICSI outcomes in both groups indicates that the cycles are comparable in terms of fertilization despite differences in cumulus gene expression. Despite low PTX3 and VDR in the PCOS group, ICSI results were similar to the control group, which led us to suspect the role of VDR and PTX3 in ovulation and fertilization. However, the report that although mouse oocytes were fertilized in vitro in the experimental Ptx3-/- model [41], in vivo fertilization did not occur, strengthened our opinion that cumulus gene defect is a pathology that can be treated with ICSI. When the results of our study and other studies are evaluated together, expression defect in cumulus expansion genes may contribute to PCOS-related subfertility by preventing in vivo fertilization, but failed expression in cumulus cell can be overcome by ICSI. As a result, we can suggest that CC-PTX3 or CC-VDR expression defect does not negatively affect oocyte developmental capacity.

​There are few studies on PTX3 expression in PCOS, and they are heterogeneous in terms of patient selection criteria, samples used for PCR, and results obtained. Pan et al. [14] reported that PTX3 protein expression in the follicular fluids of 102 PCOS patients who underwent in vitro fertilization showed a significant increase compared to normal reponders. Unlike our study, patients using both the GnRH long agonist protocol or GnRH antagonist protocol were evaluated together in that study. In addition, unlike ours, hCG used to induce ovulation in studies has a high potential to increase CC-PTX3 expression [33]. The significant increase in PTX3 expression after hCG injection in animal studies supports our idea [33, 41, 42]. In addition, PTX3 levels may have been found to be high because follicular fluid collected from only one mature follicle in each patient was evaluated. In our study, only PCOS patients who underwent GnRH antagonist protocol were included in the study. We excluded the effect of hCG on the CC-PTX3 mRNA expression, since we triggered ovulation with a GnRH agonist. Unlike Pan et al. [14], we used cumulus cells, not follicle fluid, for PCR. They also studied PTX3 protein expression while we studied PTX3 mRNA expression. The most important difference between the two studies is that we classified the cumulus cell samples according to the nuclear maturation stage. If all collected COCs were graded according to nuclear maturation stage and then follicular fluid PTX3 levels were measured, the results might have been different. Another study that analyzed CC-PTX3 expression in PCOS by nuclear maturation stage was conducted by Ouandaogo et al. [18]. These authors detected down-regulation in the expression of PTX3 and other cumulus expansion genes by microarray method in cumulus cell samples collected from PCOS patients undergoing in vivo or in vitro maturation. In that study, patients who underwent both agonist and antagonist protocols were included in the study and hCG was used for ovulation trigger. Both our study and that of Quandaogo et al. [18] are important in emphasizing that PTX3 expression of CCs varies according to the oocyte maturation stage. Although the study of Ouandaogo et al. [18] is methodologically similar to our study, the two studies differ in terms of the induction protocol used, ovulation trigger type, and cumulus cell sources. Unlike our study, the authors performed transcritomic analysis on CCs obtained from both in vitro maturation and in vivo maturation cycles. Since our study shows the changes in mRNA expression as fold changes according to the nuclear maturation stage, it allows us to more clearly interpret the change in CC-PTX3 expression between GV, MI and MII oocytes. In addition to clinical studies, expression changes of genes responsible for cumulus expansion have also been demonstrated in experimental PCOS studies. Consistent with this, a PCOS mouse model study reported that CC-PTX3 expression was increased by LH and testosterone and that metformin, an endoplasmic reticulum stress inhibitor, reversed this effect [43]. When studies evaluating cumulus cell or follicular fluid PTX3 expression in PCOS patients and our study are evaluated together, it is noteworthy that PTX3 results differ between studies. Heterogeneity of participants, differences in biological tissues or fluids used in PCR, and differences in ovarian stimulation and ovulation trigger methods may be the reasons for the different PTX3 results. It will be possible to reach clearer results with studies investigating both CC and follicular fluid PTX3 levels in PCOS patients divided into phenotypes according to the NIH 2012 consensus panel and applied to a single induction protocol.

Being a nuclear receptor, VDR is expressed in both human and animal granulosa cells, and its expression increases in proportion to the diameter of the follicle [27, 44]. Increased expression of VDR, steroidogenic acute regulator and 3β-HSD has been reported in cultures of GCs treated with VD. Genomic or non-genomic activation of the nuclear VDR increases the release of cAMP, estradiol and progesterone from GCs, while reducing the expression of anti-mullerian hormone and FSH receptor [28, 44]. Our study is the first to analyze VDR mRNA expression in CCs of PCOS patients. We found a significant increase in CC-VDR mRNA expression from GV stage oocyte to MII stage oocyte in normal responders. CC-VDR mRNA expression was significantly higher in MI and MII oocytes of normal responders than in GV oocytes. In addition, CC-VDR mRNA expression in MII oocytes of the normal responders showed a significant increase compared to MI oocytes. In PCOS group, while CC-VDR mRNA expression of MI and MII stage oocytes was significantly higher than that of GV oocytes, we did not detect a significant difference between MI and MII oocytes in terms of CC-VDR mRNA expression. In addition, the increase in CC-VDR mRNA expression betwween nuclear stages in the PCOS group was less pronounced than in normal responders. The fact that the increase detected in CC-VDR mRNA expression in transition from GV stage to MI and MII is less pronounced than in the control group, and the CC-VDR mRNA expression of oocytes transitioning from MI stage oocyte to MII is similar, suggesting that CC-VDR mRNA expression may be defective in PCOS.

By characterizing the expression profiles of VDR and PTX3 mRNA in cumulus cells obtained from PCOS oocytes at different stages of nuclear development, we had the opportunity to test the variation in expression of these two genes depending on the nuclear maturation stage. The fact that the control group consisted of non-PCOS normal responders enabled us to understand whether metabolic and hormonal changes specific to PCOS have an effect on CC-VDR and PTX3 mRNA expression. Demonstration of altered GC gene expression in individuals supplemented with Vitamin D supports the role of VD on CC-VDR mRNA expression [45, 46]. The lower serum VD levels in PCOS patients compared to normal responders may explain the defective expression of CC-VDR mRNA in the PCOS group. Low VD levels in PCOS patients with a high prevalence of implantation failure supports our idea that CC-VDR expression defect may play a role in PCOS-related subfertility [16].

Cumulus expansion is the end-stage maturation process of the oocyte that occurs under the control of multiple cumulus-derived genes and oocyte-derived factors. Hyaluronic synthase 2, gremlin1, prostaglandin synthase 2, versican, tumor necrosis factor alpha-induced protein 6 are some of the genes mainly related to the expansion, apoptosis and glucose metabolism of cumulus cells [47]. ​ In the current study, we examined the expression levels of the PTX3 gene, which is responsible for cumulus expansion, and the VDR gene, which contributes to the intra-follicle redox balance required for expansion. However, it is not possible to reach a clear discussion about ovulation dysfunction and fertilization problems in PCOS by selecting two genes from a set of genes. To date, no single cumulus gene has been found that can predict embryo quality and implantation potential. The main reason for our focus on PTX3 and VDR is to establish the existence of a single gene that can predict oocyte developmental capacity in PCOS patients. However, the similarity of fertilization rates between PCOS and normal responders suggests that the expression defect in these genes does not negatively affect oocyte developmental capacity. In summary, although a relatively limited number of cumulus cell samples were studied, our study enabled us to obtain the following gains. We demonstrated for the first time the defective expression of these two genes in PCOS patients. Secondly, we observed that VDR and PTX3 expression defect did not cause a significant change in COC morphology. Third, and most importantly, performing ICSI on oocytes that defectively expressed these two transcripts resulted in fertilization rates similar to normal responders.

The question of whether the drugs used in the antagonist protocol have an effect on the difference in PTX3 and VDR expression between PCOS and normal responders seems to be a logical limitation. High PTX3 expression in porcine cumulus cells exposed to in vivo hCG stimulation or in vitro FSH/LH treatment supports our concern [33]. However, since we used GnRH agonist instead of hCG as the ovulation trigger, we can consider that the changes in the expression of both genes occur independently of the ovulation triggering agent. The fact that the use of different doses of agonists for ovulation trigger did not change the granulosa cell gene profile strongly suggests that the differences in PTX3 and VDR mRNA expression are due to the underlying pathologies, not the drugs used in the GnRH antagonist protocol [48].

## Conclusion

CC-PTX3 and CC-VDR mRNA expressions of both PCOS and normal responders are affected by the degree of oocyte nuclear maturation. However, the relative increase in expression of both transcripts during oocyte nuclear maturation stages in PCOS is less pronounced than in normal responders. Since all patients had already undergone the COS protocol, it is possible that the failed response to upregulate the gene expressions studied here was responsible for the failure in oocyte maturation. Since defective expression of both VDR and PTX3 mRNA disrupts the organization and structural components of CCs, it may lead to CC expansion defect and failed fertilization in PCOS [7, 28]. However, the fact that fertilization rates were similar between the two groups strongly suggests that ICSI overcomes the expression defect in cumulus genes and that the fertilization problem is an in vivo process. Metabolic and hormonal pathologies specific to PCOS, as well as chronic low-grade inflammation, can impair the expression of cumulus expansion genes and oocyte developmental competence. The fact that serum VD, total testosterone and BMI values are different in the PCOS group than in normal responders supports our idea. Both VDR and PTX3, as two anti-inflammatory genes, may play a role in maintaining the pro-anti-inflammatory redox balance in cumulus expansion [40, 46]. Defective expression of both transcripts may contribute to PCOS-associated ovulatory dysfunction. Medical treatments aimed at correcting expression defects of PTX3, VDR and other cumulus genes may emerge as a new approach to the treatment of PCOS-related infertility.

## Data Availability

The datasets used and/or analyzed during the current study are available upon reasonable request with approval from the corresponding author and all other authors.
